# Assessment of Structural Connectivity in the Preterm Brain at Term Equivalent Age Using Diffusion MRI and T_2_ Relaxometry: A Network-Based Analysis

**DOI:** 10.1371/journal.pone.0068593

**Published:** 2013-08-07

**Authors:** Kerstin Pannek, Xanthy Hatzigeorgiou, Paul B. Colditz, Stephen Rose

**Affiliations:** 1 The University of Queensland, School of Medicine, Brisbane, Australia; 2 The University of Queensland, Queensland Cerebral Palsy and Rehabilitation Research Centre, Brisbane, Australia; 3 The University of Queensland, Perinatal Research Centre, Brisbane, Australia; 4 The University of Queensland and Royal Children's Hospital, Children's Nutrition Research Centre, Brisbane, Australia; 5 The University of Queensland, Centre for Clinical Research, Brisbane, Australia; 6 The Australian e-Health Research Centre, CSIRO, Brisbane, Australia; Beijing Normal University, China

## Abstract

Preterm birth is associated with a high prevalence of adverse neurodevelopmental outcome. Non-invasive techniques which can probe the neural correlates underpinning these deficits are required. This can be achieved by measuring the structural network of connections within the preterm infant's brain using diffusion MRI and tractography. We used diffusion MRI and T_2_ relaxometry to identify connections with altered white matter properties in preterm infants compared to term infants. Diffusion and T_2_ data were obtained from 9 term neonates and 18 preterm-born infants (born <32 weeks gestational age) at term equivalent age. Probabilistic tractography incorporating multiple fibre orientations was used in combination with the Johns Hopkins neonatal brain atlas to calculate the structural network of connections. Connections of altered diffusivity or T_2_, as well as their relationship with gestational age at birth and postmenstrual age at the time of MRI, were identified using the network based statistic framework. A total of 433 connections were assessed. FA was significantly reduced in 17, and T_2_ significantly increased in 18 connections in preterm infants, following correction for multiple comparisons. Cortical networks associated with affected connections mainly involved left frontal and temporal cortical areas: regions which are associated with working memory, verbal comprehension and higher cognitive function – deficits which are often observed later in children and adults born preterm. Gestational age at birth correlated with T_2_, but not diffusion in several connections. We found no association between diffusion or T_2_ and postmenstrual age at the time of MRI in preterm infants. This study demonstrates that alterations in the structural network of connections can be identified in preterm infants at term equivalent age, and that incorporation of non-diffusion measures such as T_2_ in the connectome framework provides complementary information for the assessment of brain development.

## Introduction

Preterm birth constitutes a significant health burden, with long term sequelea including cerebral palsy, mild motor impairments, cognitive deficits and educational difficulties [1], [2]. Magnetic Resonance Imaging (MRI) can be used to non-invasively image the infant's brain even before term equivalent age, and can potentially be used as an early diagnostic tool for identifying perturbed neurodevelopment [3]. Several MRI-derived indices, including fractional anisotropy (FA) and the transverse relaxation time T_2_, have been shown to be affected by the on-going organisation of white matter (WM), premyelination and myelination seen in preterm and term infants in the first years of life (e.g. [4]–[6]).

Diffusion MRI is particularly suited to studying brain development because of its ability to probe the random motion of water molecules, with resultant diffusivity measures shown to be influenced by on-going maturation in early life, including typically increased fractional anisotropy (FA) and decreased mean diffusivity (MD) with increasing age [7]. In addition to providing metrics that may reflect ongoing brain maturation, diffusion MRI also offers a method for the delineation of white matter pathways *in vivo* with tractography. Diffusion tractography studies of the preterm infant brain have predominantly assessed the corticospinal tract [8]–[17], the corpus callosum [13]–[20], and the optic radiations [11]–[13], [15], [21]–[25]. Tractography can, however, also be used to study *networks* of connections (i.e. the connectome) on the macroscale. Ball et al [24] recently demonstrated microstructural differences in the thalamocortical connectome between preterm and term infants scanned at term age; no study has, however, assessed the *cortico-cortical* network of connections in preterm infants.

The aim of the current study was to assess microstructural differences of cortico-cortical connections in preterm infants scanned at term age compared to their term-born peers, as well as to investigate the effect of the degree of prematurity and age at scan. White matter microstructure was assessed using the diffusion metrics FA and MD, and the spin-spin relaxation time T_2_. T_2_ is a non-diffusion metric that is believed to reflect myelination and water content. Region-of-interest (ROI) analyses have previously shown a decrease in T_2_ with increasing age in pediatric populations, consistent with decreases in the brain's free water content and increases in myelination [4], [6]. We therefore included T_2_ as a marker of microstructure in addition to the typically assessed diffusion metrics FA and MD.

Connections between 24 cortical regions per hemisphere were automatically delineated using whole-brain probabilistic tractography based on a diffusion model that incorporates crossing fibres [26]. Median FA, MD and T_2_ were extracted for every connection, and statistical analysis was performed using the network-based statistic (NBS; [27]), a well-established tool for identifying network components of altered connectivity [28]–[31].

## Methods

### Ethics Statement

This study was approved by the Royal Brisbane and Women's Hospital Human Research Ethics Committee (RBWH HREC 2004/149). Informed written consent was obtained from the parents of each participant.

### Participants

Twenty-five preterm (15 male, gestational age at birth 25^+3^–32^+6^ weeks) and 10 healthy term-born infants (4 male, gestational age at birth 37^+5^–40^+6^) were recruited to this study. Infants with cortical or white matter injury, haemorrhage, brain malformation, congenital infection or anomalies were excluded. MRI data were acquired within 1 week of term-equivalent age for preterm infants, and at a mean age of 3 days for term neonates.

### Data acquisition

MRI data were acquired during natural sleep without sedation using a 1.5T Siemens Sonata scanner (Siemens, Erlangen, Germany) with a quadrature knee coil. Neonatal earmuffs were used for hearing protection, and infants were placed in a vacuum fixation pillow to minimize motion. Infants were monitored visually and by pulse oximetry throughout the imaging session. Diffusion weighted images were acquired along 44 non-collinear directions at b = 1100 s/mm^2^ [32], along with 16 images without diffusion weighting using a single-echo EPI sequence. Imaging parameters were: 45 axial slices with 2.5 mm thickness and 0.25 mm slice gap; TR/TE 6000/106 ms; FOV 23×23 cm; acquisition matrix 128×128 reconstructed to 256×256. Acquisition time for the diffusion weighted data was 8.5 minutes. T_2_ MRI data were acquired using a multi-echo, fast spin echo sequence with the following parameters: 50 axial contiguous 2 mm slices, TR/(TE) 10,000/(26/128/192) ms, FOV 18×18 cm, acquisition matrix 256×192. Acquisition time for the T_2_ data was 6.5 minutes. The total scan time for the entire imaging protocol was 28 min.

### Diffusion preprocessing

Diffusion weighted images were processed using a combination of tools available with the FMRIB Software Library (FSL; [33]) and in-house tools. Image volumes with head movement (leading to signal dropout in individual slices or misalignment between the odd and even sub-volumes of the interleaved scan) were identified using the discontinuity index [34]. Two preterm and 1 term born infant who showed excessive head movement were excluded from further analysis. Diffusion weighted images were corrected for eddy current distortions and head movement using affine registration of the diffusion weighted volumes to the first image volume with minimal diffusion weighting (b = 0 image). The b-matrix was subsequently adjusted to account for head rotation [35], [36]. Skull stripping was performed on the first image with minimal diffusion weighting using the FSL brain extraction tool BET. Fractional anisotropy (FA) and mean diffusivity (MD) maps were calculated for each infant. Estimation of fibre orientations for tractography was performed using bedpostx [26]. Whole brain tractography was performed by seeding 50 probabilistic streamlines in the centre of each voxel within the brain volume. FSL's “probtrackx” command with “–verbose = 2” option was used to obtain coordinates of all streamline steps. FA and MD values were subsequently sampled at every streamline integration step using tri-linear interpolation. Individual streamline information was stored for further analysis. Track density images (TDI; [37]) were calculated for later use in image co-registration.

### Calculation of T_2_ maps

For each participant, T_2_ maps were obtained from the three T_2_-weighted images by first aligning all T_2_-weighted images to the T_2_-weighted image with the shortest echo time (TE = 26 ms) using rigid-body registration, followed by voxel-wise estimation of T_2_ employing a nonlinear least-squares fit using the relationship S(TE) = S_0_ exp(-TE/T_2_). T_2_ maps were co-registered with the diffusion TDI using mutual information rigid-body registration. Co-registration between T_2_ and TDI showed, on visual inspection, improved registration accuracy compared to co-registration with FA, MD or the image without diffusion weighting (b = 0 image) for our data. Following image co-registration, T_2_ values were sampled at every streamline integration step and individual streamline information was stored for further processing.

### Registration to JHU neonate space

Non-linear alignment from native diffusion space to Johns Hopkins University (JHU) neonate space [38] was achieved using a two-step approach. First, a study specific FA template was generated based on the FA maps of all infants using an iterative averaging approach with symmetric diffeomorphic registration (ANTS, [39]). The study specific FA template was then aligned with the JHU neonate single-subject FA map using symmetric diffeomorphic registration. This two-step approach resulted in successful registration of all images to the JHU FA template, whereas direct registration of individual FA maps to the JHU FA template failed in most cases. All streamlines contained within the whole brain tractograms of each participant were subsequently transformed to JHU neonate space for further processing.

### Connectome calculation

The JHU neonatal brain atlas [38] was used to define the nodes for connectivity analysis. This single subject atlas contains a total of 122 cortical and subcortical gray and white matter regions of interest. In this study, 24 cortical regions per hemisphere were used as target regions (nodes). For every participant, we tested for every streamline whether its terminals (length 10 steps, equivalent to 5 mm length in diffusion space) resided within any of the 48 cortical nodes. The number of streamlines connecting each pair of nodes was recorded in a connectivity matrix. Median FA, MD and T_2_ within the connection were also recorded for each pair of nodes. The median was chosen as a summary metric to take into account the potentially skewed distribution of values within a connection. Note that all assessed metrics were sampled in native space, such that no interpolation of the diffusion or T_2_ maps occurred. By using metrics sampled at each streamline integration step, brain areas that are visited by fewer streamlines automatically receive a lower weight compared to regions visited by a large number of streamlines. Therefore, no threshold was required to determine the volume within which to calculate median values.

**Figure 1:**
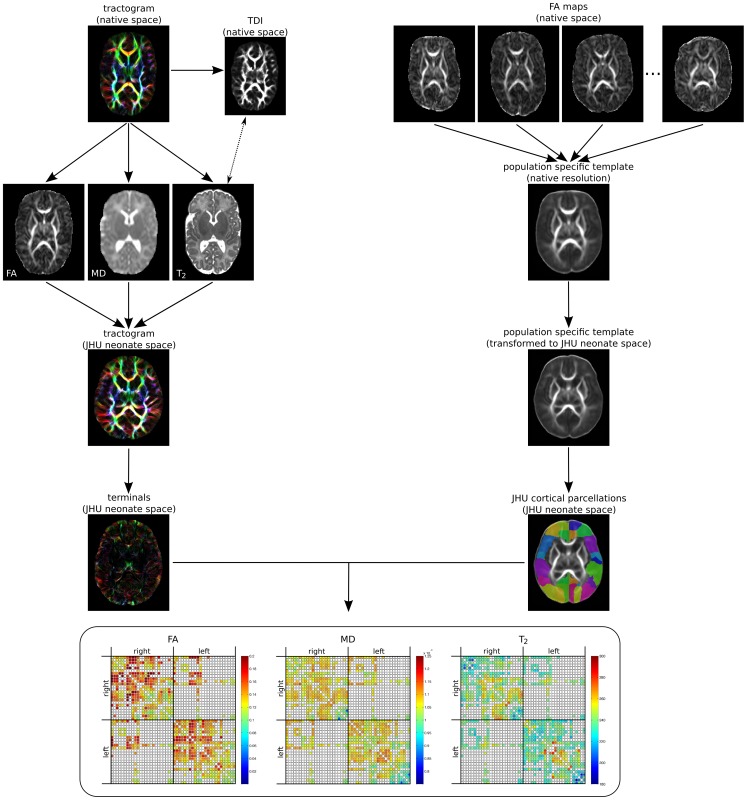
Workflow for obtaining the structural connectome. Whole brain tractograms are generated in native diffusion space. Maps of FA, MD and T_2_ (co-registered via the TDI) are sampled prior to transformation of the tractogram to JHU neonate space. Registration to JHU neonate space was performed by first calculating a study-specific FA template, which was then aligned with the JHU neonate FA template. Only the terminals of streamlines (10 points) are hit-tested with JHU neonate cortical target regions to assemble the connectomes.

Figure 1 summarises the workflow for obtaining the connectomes.

### Hypotheses

We hypothesized that (i) white matter maturation is delayed in preterm-born infants compared to their term-born peers at term equivalent age, (ii) white matter maturation is related to postmenstrual age at the time of MRI, and (iii) white matter maturation is related to the degree of prematurity. A delay in white matter maturation, i.e. reduced (pre-)myelination and organisation, was expected to be reflected by reductions in FA, and increases in MD and T_2_.

### Statistical analysis

Only connections that could be identified in all infants were included in statistical analysis. A general linear model was used (i) to identify differences in FA, MD, and T_2_ between preterm and term-born infants for every connection, with and without correcting for postmenstrual age at the time of MRI (PMA) and gender; and (ii) to identify correlations between FA, MD and T_2_ for every connection for preterm infants and gestational age at birth (GA) and PMA. Correction for multiple comparisons was performed using the network-based statistic (NBS; [27]) implemented in the NBS toolbox for Matlab (https://sites.google.com/site/bctnet/comparison/nbs). The NBS identifies statistically significant network components (i.e. “clusters” of connections), and can be thought of as the network analogue of conventional cluster statistics that is typically performed on statistical parametric maps. The NBS has a greater power than other correction methods such as Bonferroni correction or false discovery rate because it takes into account the interconnections (i.e. common nodes) between individual connections. A threshold of the t-statistic of 3 was used for individual connections as described previously [27], which were subsequently included in permutation testing. Ten-thousand permutations were generated to build up the null-distribution.

To assess how frequently each cortical region was associated with alterations in microstructure, we counted, for every cortical region, the number of connections that exhibited group difference or relationships with PMA or GA, respectively. This information was subsequently colour-coded onto a 3D rendering of the cortex.

## Results

### Demographics

Thirty-five infants (25 preterm, 10 term) met the inclusion criteria for recruitment. Data of 3 infants (2 preterm, 1 term) were excluded due to extensive head motion artefacts. Data of one preterm infant were excluded due to a corrupted diffusion encoding gradient table. In 4 preterm infants, the fibre orientations could not be calculated correctly and whole brain tractography could not be performed. The final cohort for diffusion analysis therefore consisted of 18 preterm infants (12 male) and 9 term neonates (3 male). Participant demographics are summarised in Table 1. For T_2_ analysis, another 3 preterm infants (2 male) and 4 term infants (3 male) were excluded due to motion on the T_2_ images. The final cohort for T_2_ analysis consisted of 15 preterm (10 males) and 5 term born infants (all female). For both diffusion and T_2_ analysis, the preterm cohort included more males than the term cohort. Information regarding GA at birth and birth weight were unavailable for 2 term born infants, and these infants were subsequently excluded from group analysis that used postmenstrual age at the time of MRI as a confounding variable.

**Table 1:** Demographics.

		Preterm-born		Term-born	
		diffusion	T_2_	diffusion	T_2_
n (male/female)		18 (12/6)	15 (10/5)	9 (3/6)	5(0/5)
GA at birth [weeks]	range	25^+3^–32^+6^	25^+3^–32^+6^	37^+5^–40^+6^	37^+5^–40^+6^
	mean (SD)	29.5 (2.3)	29.9 (2.5)	39.3 (1.2)	39.1 (1.6)
weight at birth [g]	range	605–1934	605–1934	2450–4250	2450–4250
	mean (SD)	1314 (371)	1327 (404)	3531 (665)	3295 (905)
Apgar (1 minute)	range	3–9	3–9	7–10	7–10
	mean (SD)	6.9 (1.9)	8.4 (2.0)	8.3 (1.2)	8.7 (1.5)
Apgar (5 minutes)	range	6–9	6–9	8–10	9–10
	mean (SD)	8.4 (0.9)	8.5 (1.0)	9.0 (1.2)	9.3 (0.6)
PMA at MRI [weeks]	range	38^+5^–45^+0^	38^+5^–45^+0^	37^+6^–41^+2^	37^+6^–41^+2^
	mean (SD)	41.3 (1.5)	41.4 (1.5)	39.6 (1.2)	39.4 (1.7)

note: data for 2 term-born infants were not available; GA: gestational age; PMA: postmenstrual age.

### NBS analysis

**Figure 2:**
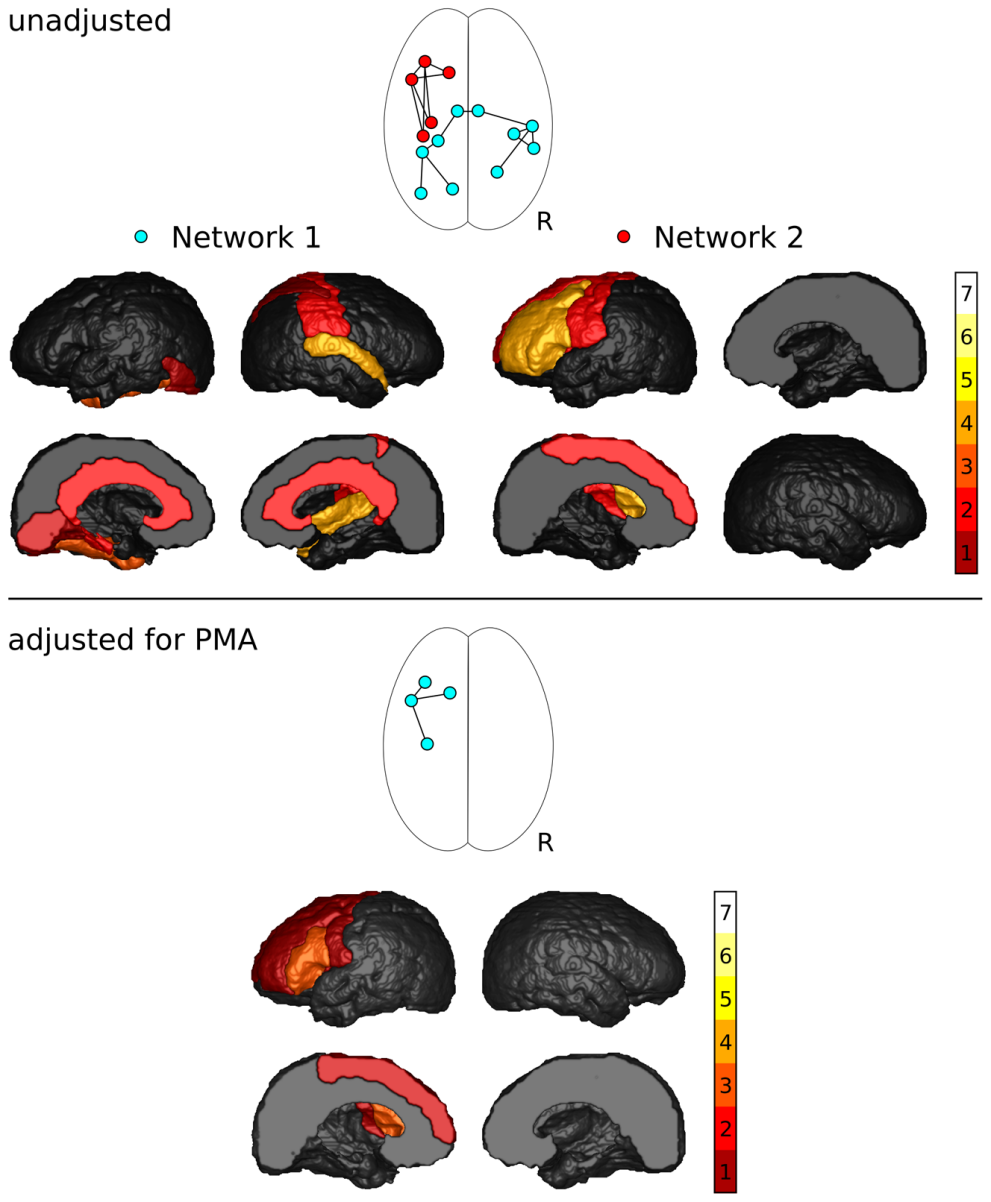
Results of the NBS analysis of FA. Shown are the network components with significantly decreased FA in preterm infants, and frequency with which cortical regions were associated with changes in FA of their connections.

**Figure 3:**
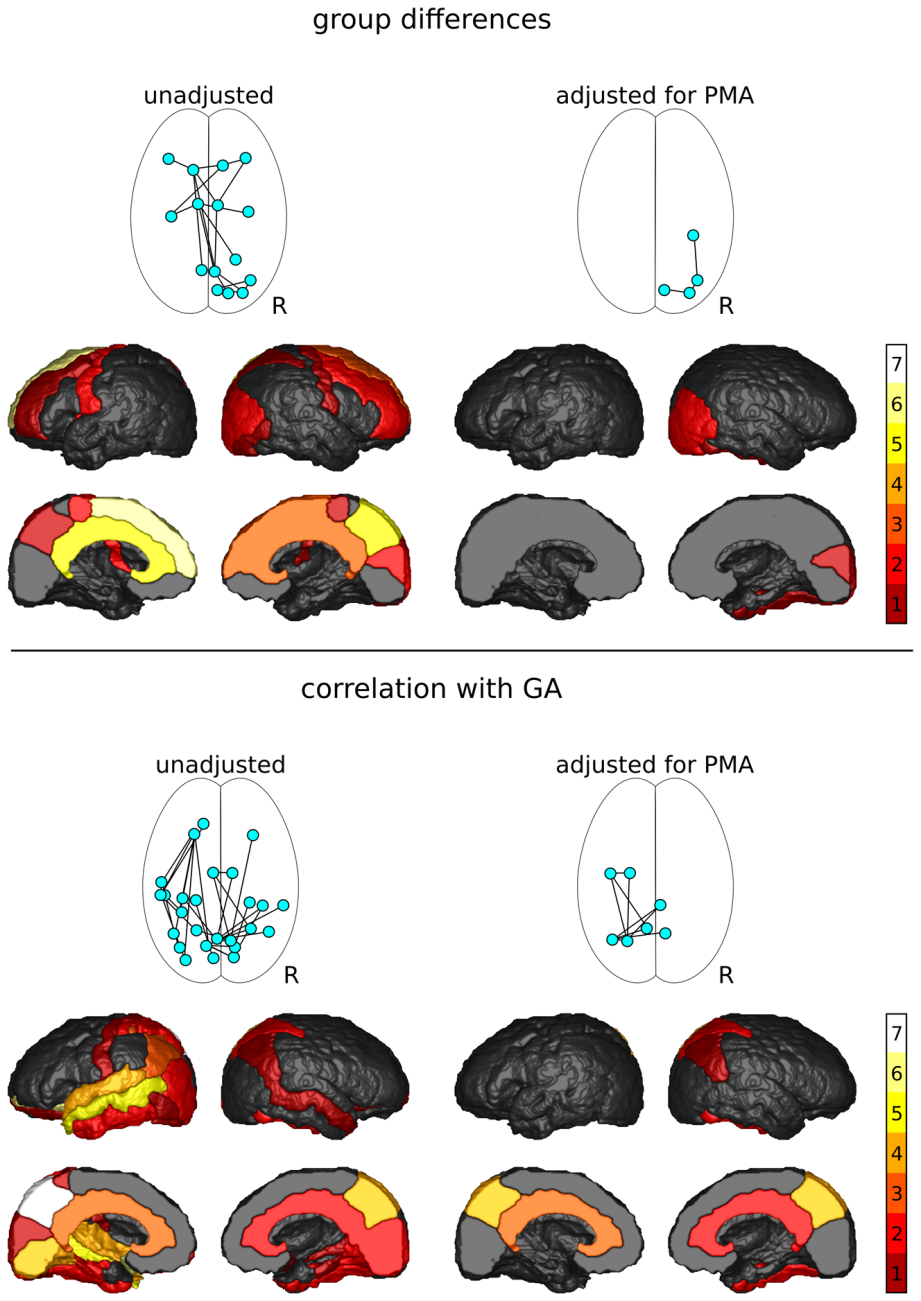
Results of the NBS analysis of T_2_. Shown are the network components with significantly increased T_2_ in preterm infants and the frequency with which cortical regions were associated with changes in T_2_ of their connections (top), and network components in which T_2_ correlated with gestational age (GA; bottom).

A total of 433 unique connections were assessed. Network components showing significant differences between groups and correlations with GA and PMA, and frequency of the identified nodes are shown in Figures 2 and 3.

#### FA group comparison

FA was significantly reduced in preterm infants for two components of the network (Figure 2, top). Component 1 comprised 10 connections between 10 nodes (p = 0.022). This component consisted of 5 right intra-hemispheric, 4 left intra-hemispheric and 1 inter-hemispheric connection. Five nodes were situated in the left, and 5 within the right hemisphere. The central nodes of this network component were the right superior temporal lobe (4 connections) and the left fusiform gyrus (3 connections). Component 2 was a left-hemisphere network consisting of 7 connections between 5 frontal and motor nodes (p = 0.0373). The central nodes of this network component were the left middle frontal lobe (4 connections) and the left interior frontal lobe (4 connections).

After adjusting for PMA, one component of the network approached, but did not reach, significance (p = 0.072; Figure 2, bottom), consisting of 4 left intra-hemispheric connections between 3 frontal and motor regions. All connections that were part of this component were also part of the second component that was identified without adjusting for PMA. The central node was the left interior frontal lobe (3 connections). After the addition of gender as potential confounder, the p-value of this component increased to 0.077.

No network components showed increased FA in preterm infants with or without adjustment for PMA and gender.

#### MD group comparison

MD was increased in preterm infants in a single, large component of the network (p<0.001), which consisted of 359 connections between 47 nodes. Following correction for PMA, component size reduced to 285 connections between 46 nodes (p<0.001). After adding gender as potential confounder, the component size reduced to 257 connections between 46 nodes (p<0.001).

No network components were identified that showed decreased MD in preterm infants with or without adjustment for PMA.

#### T_2_ group comparison

T_2_ was increased in preterm infants in one component of the network (p = 0.015; Figure 3, top). This network component consisted of 18 connections between 15 nodes, including 7 left intra-hemispheric, 7 right intra-hemispheric and 4 inter-hemispheric connections. Five nodes were in the left, and 10 in the right hemisphere. The central nodes were the left superior frontal lobe (6 connections), the left cingular gyrus (5 connections) and the right precuneus (5 connections). After adjustment for PMA, the component size reduced to 3 right hemisphere connections between 4 occipital and temporal regions. The difference in T_2_ approached, but did not reach, significance after adjustment for PMA (p = 0.059). This component no longer approached significance after gender was added as potential confounder (p>0.10).

No network components showed decreases in T_2_ in preterm infants with or without adjustment for PMA and gender.

### Correlations with PMA

There was no correlation between FA, MD or T_2_ and PMA for the preterm infant group.

### Correlation with GA

FA and MD did not correlate with GA in any network component.

A negative correlation between T_2_ and GA was found for one component of the network (p = 0.014; Figure 3, bottom). This network component consisted of 32 connections between 26 nodes, and included 3 right intra-hemispheric, 17 left intra-hemispheric and 12 inter-hemispheric connections. Ten nodes were found in the right hemisphere, and 16 in the left hemisphere. The central nodes in this network were the left precuneus (7 connections), the left lateral orbito-frontal (6 connections), the left middle temporal (5 connections), and the left superior temporal, left lingual, and right precuneus (4 connections each).

Following adjustment for PMA, a component consisting of 9 connections between 7 nodes showed a negative correlation between T_2_ and GA (p = 0.046). This component had 2 right intra-hemispheric connections and 7 inter-hemispheric connections. The central nodes in this network component were the left and right precuneus (4 connections each).

## Discussion

In this study, we used diffusion MR tractography to assess the effect of prematurity on white matter microstructure using a network approach. Importantly, we included not only diffusion metrics in the assessment of white matter development, but also incorporated T_2_ relaxation times in the connectome. The network based statistic [27] was used to identify components of the network within which FA, MD or T_2_ were altered in preterm infants compared to term infants; and to identify components of the network within which FA, MD or T_2_ correlated with GA at birth or PMA at MRI. While FA was reduced in preterm infants in a network component involving left frontal and motor areas, and T_2_ was increased in a network component involving right occipital and temporal connections, MD was increased within the majority of assessed connections.

This finding might suggest that MD is more sensitive for detecting white matter differences than FA and T_2_. On the other hand, the observed *global* increase in MD indicates that this metric may be more influenced by water content of the brain [40] rather than (pre-) myelination and organisation - processes that are expected to be affected in individual groups of pathways. We therefore speculate that, in our cohort, the differences observed in MD are predominantly influenced by water content rather than (pre-)myelination or organisation, while differences in FA reflect changes in organisation and alterations in T_2_ reflect ongoing (pre-)myelination.

Interestingly, the components in which differences between preterm and term infants were detected in both FA *and* T_2_ overlap only in a single connection (before adjustment for PMA). This finding highlights the importance of using multiple image contrasts to assess brain maturation: preterm birth may have a more pronounced effect on white matter organisation (and hence FA) in one subnetwork of the brain, and a more pronounced effect on (pre-) myelination (and hence T_2_) in another subnetwork.

Inclusion of further imaging modalities and metrics will provide a more complete picture of maturation and the effects of preterm birth on cortico-cortical connectivity.

In addition to identifying connections of altered microstructure in preterm infants compared to term infants, we investigated whether the degree of prematurity (i.e. GA at birth) influenced WM microstructure in any network components. We found that diffusion metrics were not related to GA at birth, and that T_2_ showed a statistically significant decrease with GA at birth in a network component involving primarily connections of the left and right precuneus. To our knowledge, no study has investigated the relationship between T_2_ and GA. Our finding indicates that infants born at later GA show a higher degree of (pre-)myelination, and therefore improved brain maturation, compared to infants born at earlier GA. The effect of prematurity on diffusion metrics assessed using tractography has not yet been extensively investigated; while a relationship between FA and GA was found in some pathways [17], [19], [25], no such relationship could be identified in other pathways, or even within the same pathways in different studies [10], [11], [14], [17], [19]. Our finding of alterations in FA and MD in preterm infants compared to term infants does, however, indicate that these metrics are affected by prematurity. Differences in MD (but not FA) between preterm and term infants in callosal pathways were also reported by Thompson et al [20], and differences in anisotropy and coherence were found in thalamocortical connections [24]. A larger cohort of preterm infants with a larger GA range will be required to assess the relationship between microstructure and GA.

An important factor known to affect anisotropy and diffusivity is the PMA at the time of MRI. In preterm infants, FA has been shown to increase with increasing PMA [8]–[10], [14], [17], [22], [25], while MD has been shown to decrease with increasing PMA [8]–[10], [14], [17], [22] in various WM pathways. Whilst no studies have assessed T_2_ changes with PMA within pathways delineated by tractography, ROI analyses have found a negative relationship with PMA [41] and age [4]–[6]. In our study, however, none of the assessed metrics correlated with PMA at the time of MRI. This discrepancy with previous studies is likely due to the narrow PMA range of infants included in this study with the majority of infants being scanned at 42^+0^ (+/−3 days), and the relatively small sample size.

Figures 2 and 3 reveal that the cortical regions most frequently associated with altered microstructure within their connections include the left and right precuneus, as well as the left superior, middle, inferior, and lateral orbito-frontal gyri, left middle and superior temporal regions, and left fusiform and lingual gyri - regions that are associated with higher cognitive function, working memory, language production, verbal comprehension and executive function. Deficits in these functions are often observed in children and adults born preterm [1], [2]. Previous reports investigating cortical thickness in children and adolescents born preterm have identified areas of reduced cortical thickness in the frontal lobes [42]–[44] and motor regions [42], [43] compared to their term-born peers. Some studies have also reported reduced cortical thickness in the parietal, occipital and temporal lobes [42]–[46]. At this stage, the relationship between cortical thickness and cortico-cortical connectivity is unknown. However, the agreement between reduced connectivity measures at term equivalent and reduced cortical thickness in children and adolescents shows promise for measuring early brain development using connectivity.

This study has some limitations. Only a small number of infants were assessed; assessment of a larger cohort at a wider gestational age range may have improved our ability to find correlations between diffusion metrics and gestational age at birth. Furthermore, there were more male infants in the preterm group compared to the term group, which may have influenced our results. Relatively small sample size and lack of gender balance in the final data analysed meant that we were unable to examine the relationship between gender and MRI measures. Information on the neurodevelopmental outcome of preterm infants was not available. Comparison between infants with adverse outcome compared to control infants or infants with a good outcome could have resulted in an increased number of connections identified.

In this study, we used an atlas template for identifying cortical target regions, which did not take into account the cortical folding of individual infants. The use of individualised cortical regions – such as can be obtained using Freesurfer [47] with adult MRI – would improve the accuracy of the identified connections by enabling the calculation of a termination mask to prevent streamlines from crossing cortical folds. The inclusion of subcortical target regions, such as the thalamus and brainstem/cerebral peduncles, would provide additional information about the connectivity of subcortical structures.

The occurrence of false positives (connections known not to exist anatomically) and false negatives (failure to delineate known anatomical connections) is a well known limitation of all tractography studies. The use of a crossing fibre model to identify fibre orientations for tractography, as performed in the current study, significantly reduces the incidence of false positives and false negatives. Additionally, the preprocessing protocol employed in this study helped improve the quality of diffusion data, further reducing artifactual tractography results. In several previous studies, streamline number and tract volume were chosen as measures of connectivity. However, both these measures are likely strongly influenced by head size and geometry; normalisation has not yet been sufficiently addressed and interpretation is not straightforward [48]. To overcome this issue, we used streamline number only to determine the presence of a connection by using a streamline number threshold, and used summary measures of quantitative metrics (FA, MD, T_2_) to characterise maturation of connections. The incorporation of non-diffusion quantitative metrics, such as T_2_, can provide insights into brain maturation using the connectome framework without relying merely on diffusion metrics to characterize white matter properties.

Previous studies of preterm infants using diffusion tractography have investigated only a small number of tracts, namely the corticospinal tract [8]–[17], [49], the corpus callosum [13], [15]–[20], [50], thalamic radiations [11]–[13], [15], [21]–[25], and the superior longitudinal fasciculus [11]–[13], [15]. Studies of individual tracts, similar to traditional region of interest (ROI) analysis, require strong *a priori* hypotheses regarding affected tracts. While whole-brain voxel based analysis is typically used to overcome this limitation of ROI analysis, in tractography studies this limitation can be overcome by analysis of the network of connections, as performed in the current study. With this type of analysis, “clusters” of connections with altered microstructure can be identified. In a different approach, Ball and colleagues successfully assessed the thalamocortical aspect of the connectome in preterm infants [24]. In our study, we have shown that a network approach can also be used to investigate brain development of cortico-cortical connections in preterm infants at term age.

In conclusion, we used diffusion tractography to assess differences in microstructure – as measured by altered FA, MD and T_2_ – in preterm-born infants compared to term-born neonates at term equivalent age. Our results indicate that, while both diffusion and relaxation time measures identify connections of altered WM properties between these participant groups, only relaxation time measures were associated with the degree of prematurity, indicating that diffusion and relaxation time provide complementary information about different processes occurring during early development.
